# Thrombus Extraction Catheters vs. Angiojet Rheolytic Thrombectomy in Thrombotic Lesions/SV Grafts

**DOI:** 10.2174/157340312803217265

**Published:** 2012-08

**Authors:** Dimitrios Alexopoulos, Periklis A Davlouros

**Affiliations:** Cardiology Department, Patras University Hospital, Patras, Greece

**Keywords:** Thrombectomy, percutaneous coronary intervention, vein grafts.

## Abstract

Primary percutaneous coronary intervention, (pPCI), of native coronaries and saphenous vein grafts (SVGs), is the recommended reperfusion strategy for STEMI, and an early invasive approach is recommended for high risk patients with UA/NSTEMI. Although PCI effectively restores flow in the infarct related artery/culprit vessel in both situations, myocardial perfusion often remains suboptimal due to microvascular obstruction, partly attributed to distal embolization of thrombus. Hence, thrombectomy (manual or mechanical), prior to stenting may further reduce hard clinical end points in patients with ACS. This article discusses accumulated evidence regarding the safety and effectiveness of thrombectomy in culprit native coronaries and SVGs in such patients, as well as possible strategies for maximizing its benefits relative to the size of the thrombotic burden.

## INTRODUCTION

**Fig. (1):**
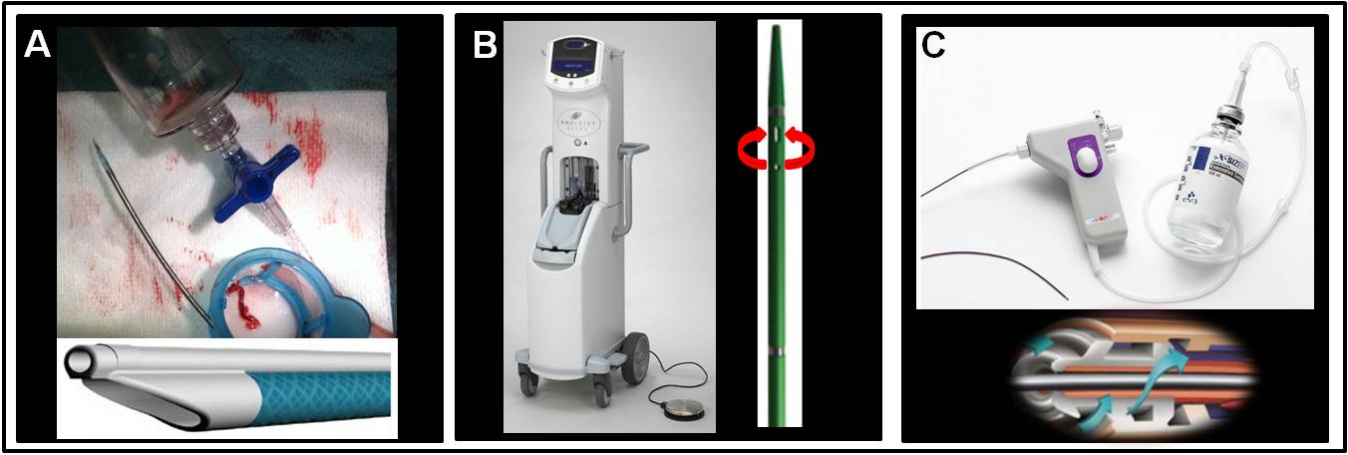
**Panel A:** The Export thrombus aspiration catheter (Medronic Vascular, USA) is a monorail system consisting of a dual lumen (see bottom insert) one for advancement over the wire (upper lumen) and one for thrombus aspiration (lower large lumen), with a distal radiopaque tip marker and a proximal luer lock port attached to a syringe for application of hand-powered suction to remove thrombus. **Panel B:** The AngioJet Ultra Thrombectomy System (Medrad Interventional/Possis, Medical, Minneapolis, Minnesota). Main unit on the left, catheter on the right. Mechanical thrombectomy is achieved by injecting pressurized saline through a hypotube by the distal tip of the coronary catheter (red arrows), thereby leading to a low-pressure zone (Bernoulli effect). The latter fragments the thrombus and the resulting debris is aspirated back and removed (red arrows). **Panel C:** The X-Sizer system (eV3, White Bear Lake, Minnesota). The X-SIZER device consists of a helical cutter rotated at 􀀁2,100 rpm, which entrains and macerates thrombus and soft plaques but not fibrocalcific tissue. The drive unit, catheter shaft, aspiration tubing and vacuum bottle are shown, with a schematic representation of the helical cutting tip (bottom insert).

Contemporary management of patients with ST-elevation myocardial infarction, (STEMI), with primary angioplasty, (pPCI), is the recommended reperfusion strategy for STEMI [1]. In high risk patients with non-STEMI acute coronary syndromes (ACS), an early invasive approach is also strongly recommended [2]. Although PCI restores flow in the infarct related artery in most patients with ACS, myocardial perfusion often remains suboptimal due to microvascular obstruction, partly attributed to distal embolization of thrombus [3, 4]. This in turn, is associated with larger infarct size, increased early and late mortality, and higher rates of arrhythmia and heart failure. Hence, devices designed to remove thrombus (like manual aspiration or mechanical thrombectomy catheters), or prevent distal embolizaton, have been developed during the last 10 years (Fig. **1**).

## ASPIRATION THROMBECTOMY (AT)

**Fig. (2):**
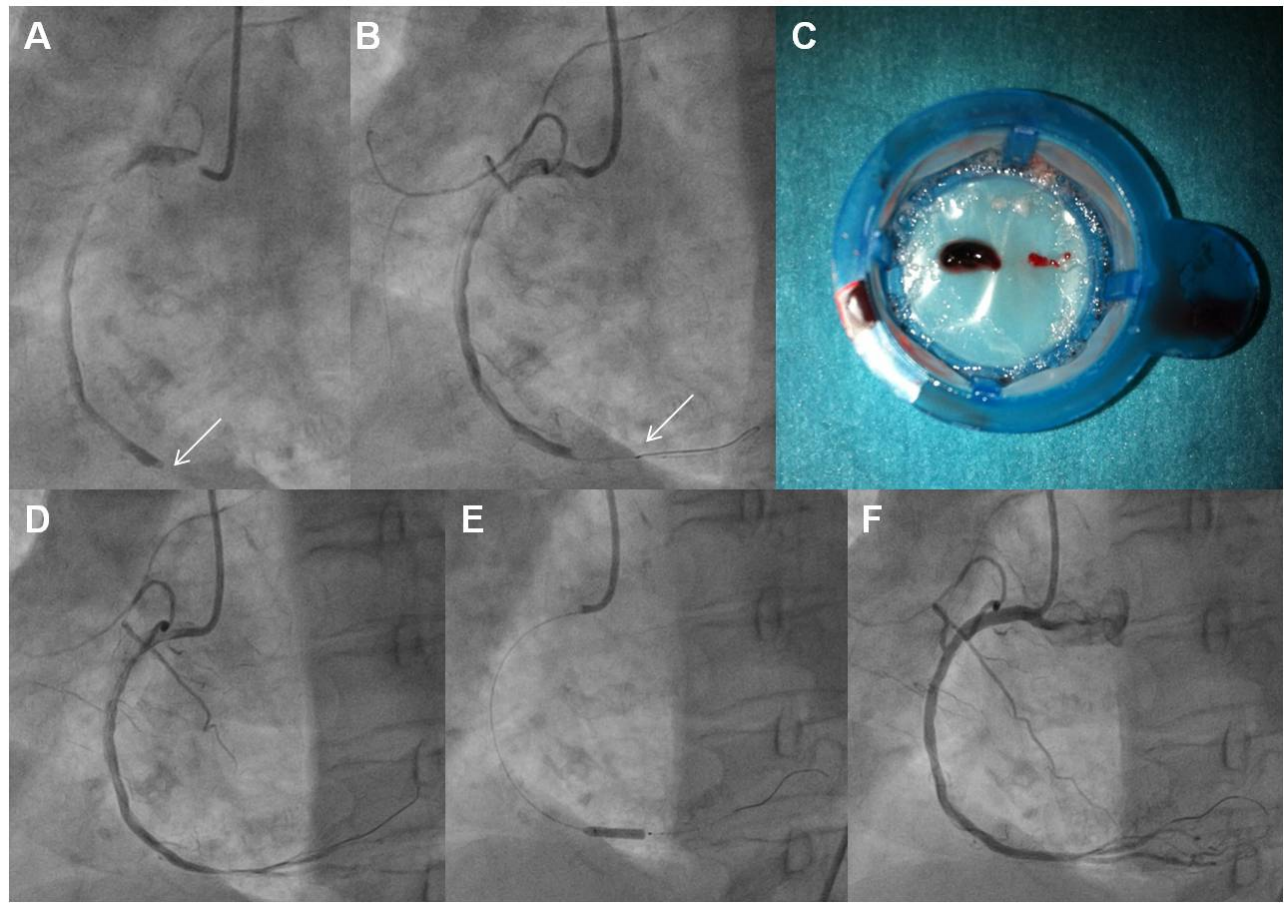
**Panel A:** A totally occluded RCA (arrow) in a patient with STEMI. **Panel B:** The distal radiopaque tip (arrow) of an aspiration catheter advanced through the lesion this shown. **Panel C:** Thrombotic material extracted. **Panel D:** Angiographic appearance of the artery following thrombus extraction. **Panel E:** Stenting of the lesion. **Panel F:** Final angiographic result.

**Fig. (3):**
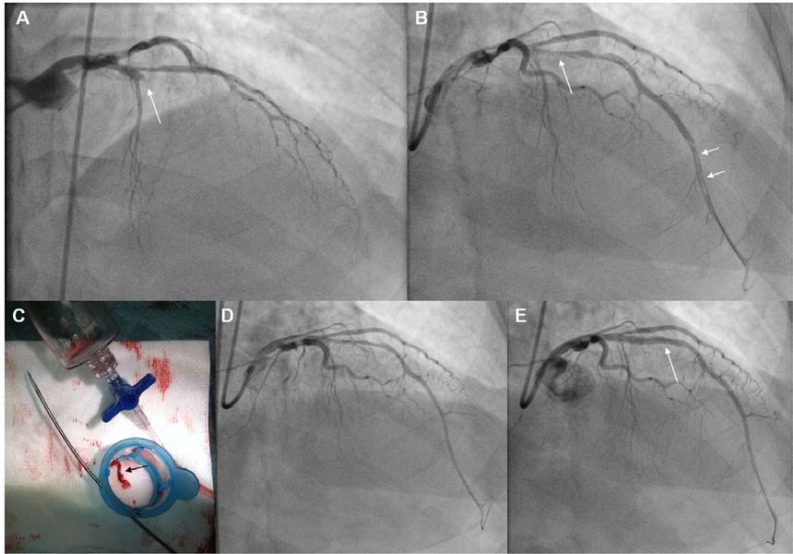
**Panel A:** Angiography (RAO cranial projection), of a patient with an anterior STEMI. The LAD is totally occluded (TIMI-0 flow) at its proximal part (arrow). Panel B: Following crossing with the guidewire and Dottering with a 1.5 mm balloon, the full length of the LAD was opacified, allowing visualization of the lesion (single arrow), and of a long filling defect corresponding to thrombus at the distal part of the artery (double arrows). **Panel C:** The Export thrombus aspiration catheter (Medronic Vascular, USA) was advanced through the lesion up to the filling defect, and following multiple passages a long thrombus was extracted (black arrow). This catheter is a monorail system consisting of a dual lumen (double arrows) one for advancement over the wire (upper arrow) and one for thrombus aspiration (lower arrow), with a distal radiopaque tip marker and a proximal luer lock port attached to a syringe for application of hand-powered suction to remove thrombus. **Panel D:** The filling defect in distal LAD has disappeared following thrombus removal. There is diffuse vasospasm due to device passage through the vessel, with TIMI-II flow. **Panel E:** After intracoronary administration of nitroglycerine and stenting of the LAD lesion (arrow), TIMI-III flow was restored. LAD: Left Anterior Descending artery.

Aspiration catheters usually consist of a monorail dual lumen system with a distal radiopaque tip marker and a proximal luer lock port attached to a syringe for hand-powered suction to remove thrombus. (Figs. **1**, **2**, **3**). Manual thrombectomy is simple, and is generally considered safe when performed according to a standard technique, which includes avoiding balloon pre-dilatation, aspirating with initial antegrade advancement of the catheter, and performing multiple passages until disappearance of visible thrombus [5].

The ESC Guidelines on myocardial revascularization list AT during pPCI in STEMI, as a Class IIa, level of evidence-A, indication [6], whereas a Class IIa indication with level of evidence-B, is listed in the AHA/ACC Guidelines on STEMI management [7, 8]. These recommendations resulted from a large number of studies with various devices showing mainly an improvement in surrogate procedural end-points. The EXPIRA study, compared the Export aspiration catheter (Medronic Vascular, USA), versus pPCI alone, and showed that the former resulted in a significant improvement of myocardial blush grade (MBG) and complete ST-segment elevation resolution (STR) [9]. This small study (175 patients), involved MRI imaging and showed that the extend of microvascular obstruction was less in the acute phase with aspiration, leading to a smaller infarct size at 3 months. The landmark TAPAS study in patients with STEMI, found that AT with the Export catheter resulted in improved myocardial reperfusion and more frequent complete STR, compared with conventional PCI [10]. Aspiration was able to be performed in 90% of patients and was successful (judged by histopathological evidence of atherothrombotic material) in 72.9%. Patients with better MBG had fewer adverse events at 30 days, and this was regarded as indirect evidence of the beneficial role of AT [10]. Indeed, at 1 year cardiac death was significantly reduced by 46% in the AT group [11]. Despite its impressive results, TAPAS was a single centre study, not powered to detect differences in clinical endpoints [12]. Since then three large meta-analyses have consecutively shown a mortality reduction with AT compared to pPCI alone [13-15]. In contrast, a recent Bayesian meta-analysis of 16 trials showed that AT was associated with fewer distal emboli, less no-reflow, more frequent TIMI 3 flow post-PCI, more STR >50%, and more MBG-3, but no significant 30-day mortality reduction [16, 17].

## MECHANICAL THROMBECTOMY (MT)

The most frequently used MT devices are the AngioJet (Medrad Interventional/Possis, Medical, Minneapolis, Minnesota), providing rheolytic thrombectomy (RT), and the X-Sizer system (eV3, White Bear Lake, Minnesota), (Fig. **1**). With both devices, multiple passes across the lesion should be performed until optimal angiographic result. Most of the studies in native coronaries have been performed with RT. A study of 100 patients showed that initial RT was associated with a lower corrected TIMI frame count, and a smaller infarct size compared to direct stenting alone [18]. Others have also shown improvement in surrogate procedural end-points when using RT to treat stent thrombosis [19]. A larger study, the AiMI trial, enrolling 480 patients with STEMI (<12 hours) showed a lower final TIMI-3 flow, and higher infarct size and 30-day MACE in the RT group compared with pPCI alone [20]. This disappointing result was attributed to the extremely low mortality rate in the PCI alone arm of the study and to technical details. Balloon pre-dilatation was usually performed before RT, and the AngioJet catheter was advanced beyond the occlusion before activation, which both may have promoted distal embolization. A Bayesian metaanalysis comparing RT (11 studies, 1.018 patients) versus PCI (81 studies, 2.076 patients) in AMI, found similar odds of short-term mortality, MACE, and TIMI-3 flow, despite the higher risk profile of the RT group (larger thrombus, more rescue PCIs, longer symptom duration) [21]. In the rescue PCI subgroup, RT demonstrated increased TIMI-3 flow postprocedure and lower mortality compared to pPCI. Hence, adjunctive RT might be beneficial in high risk subgroups of STEMI patients e.g. those with large thrombus [21]. In accord with this, RT compared to PCI alone in patients with large thrombus resulted in higher TIMI flow, MBG, and absence of thrombus, which was associated with a higher 2-year cumulative survival and MACE-free survival [22]. Another study showed that STEMI patients with moderate-to-large thrombus treated with RT compared to pPCI alone had better TIMI flow post-PCI with less no-reflow, better MBG, and a trend towards lower 30-day and 1-year MACE [23]. The recent JetSTENT trial in 501 STEMI patients with large thrombus burden (TB), showed better STR and lower MACE at 6 months and 1 year with RT versus direct stenting alone [24]. The difference in clinical outcome was driven by death and target vessel revascularization. This trial was very well designed and conducted, with inclusion only of patients with angiographic evidence of moderate-large thrombus, or an occluded artery following coronary wiring. Activation of the RT catheter was done proximal to the thrombus and a single pass was performed to decrease the risk of embolization. In contrast to the TAPAS and AiMI trials, in the JETSTENT trial, routine direct stenting without pre- or postdilation was applied in both arms (thrombectomy vs. stenting), along with routine use of abciximab in both arms. Thus the results of the JETSTENT trial have revived the concept of RT especially for AMI patients with large TB. Non-randomized registry data further reinforced the concept of benefit with RT in STEMI patients with large TB, as it was associated with reduced distal embolization, thereby improving MBG and TIMI flow, which led to a trend toward lower 1-year mortality [25]. These promising new data regarding RT have not been incorporated in the guidelines for PCI and/or STEMI [6-8]. Besides needing an increased procedural time, RT is considered relatively safe, however care must exerted in case of severe tortuosity, especially in small vessels (< 2.0 mm), to avoid disruption of the artery [25]. Hemodynamically significant AV block may complicate the procedure, especially when the RCA (or dominant left circumflex) is involved, thus some operators recommend placement of a temporary pacemaker, although temporary pacemakers were not used in the JetSTENT Trial [24]. Specific technical precautions include avoiding balloon predilatation, activating the device at least 1 cm proximal to the thrombus before advancing beyond the lesion, advancement speed of 1-3 mm per second, and keeping the device activated during multiple passages until all visible thrombus disappears [26].

## TESTING THE STRATEGIES: ASPIRATION VS. MECHANICAL AND UNIVERSAL VS. SELECTIVE

### Thrombectomy

Most studies and meta-analyses -until recently- tend to concur that the benefit of thrombectomy in STEMI appears to be dependent on the type of technique used, with no clinical benefit for routine MT. However, no direct comparison of the two techniques exists.

A meta-analysis of 27 trials using showed benefit of AT and possible harm of MT during pPCI for STEMI [13]. This result seems to have been driven by the AiMI study. Similarly, a meta-analysis of 11 trials using a patient level analysis showed that only STEMI patients treated with AT -and not MT- experienced a mortality and MACE reduction, especially if glycoprotein IIb/IIIa inhibitors were used [15]. This meta-analysis included only two small studies of MT, using AngioJet [18], and X-Sizer [27]. Another two studies registered as MT in this meta-analysis, used the TVAC, (Nipro's TransVascular Aspiration Catheter, Osaka, Japan), which is a single lumen aspiration catheter with a dedicated vacuum pump [28], and the Rescue catheter (Boston Scientific), which is an aspiration catheter [29]. On the contrary, the latest Bayesian meta-analysis of 16 AT trials and 5 MT trials (4299 patients), that reported separately the aspiration results, showed that thrombectomy in general is associated only with improved surrogate procedural end-points, with equipoise results between AT and MT [16]. The JetSTENT trial that followed the above meta-analyses, revived interest in RT [24]. It may be that these two techniques (AT vs. MT) have differential benefits relative to the angiographic situation applied. For example, AT is not always successful, especially in patients with large thrombus, and may also promote by itself distal embolization and no-reflow [29], whereas RT is very effective in removing large thrombus [21-24, 26]. Therefore, large studies comparing the two techniques, possibly under different angiographic scenarios are needed. Until then, AT remains the recommended technique for adjunctive thrombectomy in STEMI [6-8].

Most thrombectomy studies have compared thrombectomy with PCI in an all-comers basis. A study with negative results (larger infarct size), using the Rescue catheter as routine therapy in 215 patients with STEMI, concluded that thrombectomy should not be used in unselected pPCI patients [29]. This, combined with recent evidence on better results of RT in patients with large thrombus burden (TB) [21-24], and cost considerations, raise the issue of defining certain subgroups of patients which may benefit the most from thrombectomy. As large TB (visible thrombus size ≥ 2 vessel diameter), during pPCI, is associated with adverse procedural and clinical outcomes compared to small TB [30, 31], it has been proposed that patients with higher TB grade might benefit the most from thrombectomy. This concept, has been tested only in recent studies using RT [21-24]. 

The largest study enrolling only patients with large TB is the JetSTENT trial. The positive results of the latter contrary to AiMI, were partly attributed to the selected population of high risk patients enrolled. On the other hand, there is lack of data regarding possible differential benefits of AT relative to the TB due to universal application and insufficient numbers of patients to evaluate subgroups in AT studies. Hence, application of AT only in STEMIpatients with large TB, is not officially recommended [6-8]. However, experts are proposing direct stenting for low-grade thrombus, and application of any thrombectomy selectively only in patients with visible thrombus [26]. A further segregation according to TB gradeproposes, AT for moderate thrombus and RT for high-grade thrombus. For patients with total occlusion of the vessel, (60% of STEMI patients) [31], direct use of a thrombectomy device, (Figs. **1**, **3**), or alternatively establishing reperfusion using a wire, or by Dottering, or by dilatation with a small balloon, is recommended, as it may help to reclassify up to 97% of patients [26, 31], (Fig. **2**). (Fig. **5**), shows a proposed algorithm for use of thrombectomy devices in STEMI.

## THROMBECTOMY IN SVGS

**Fig. (4):**
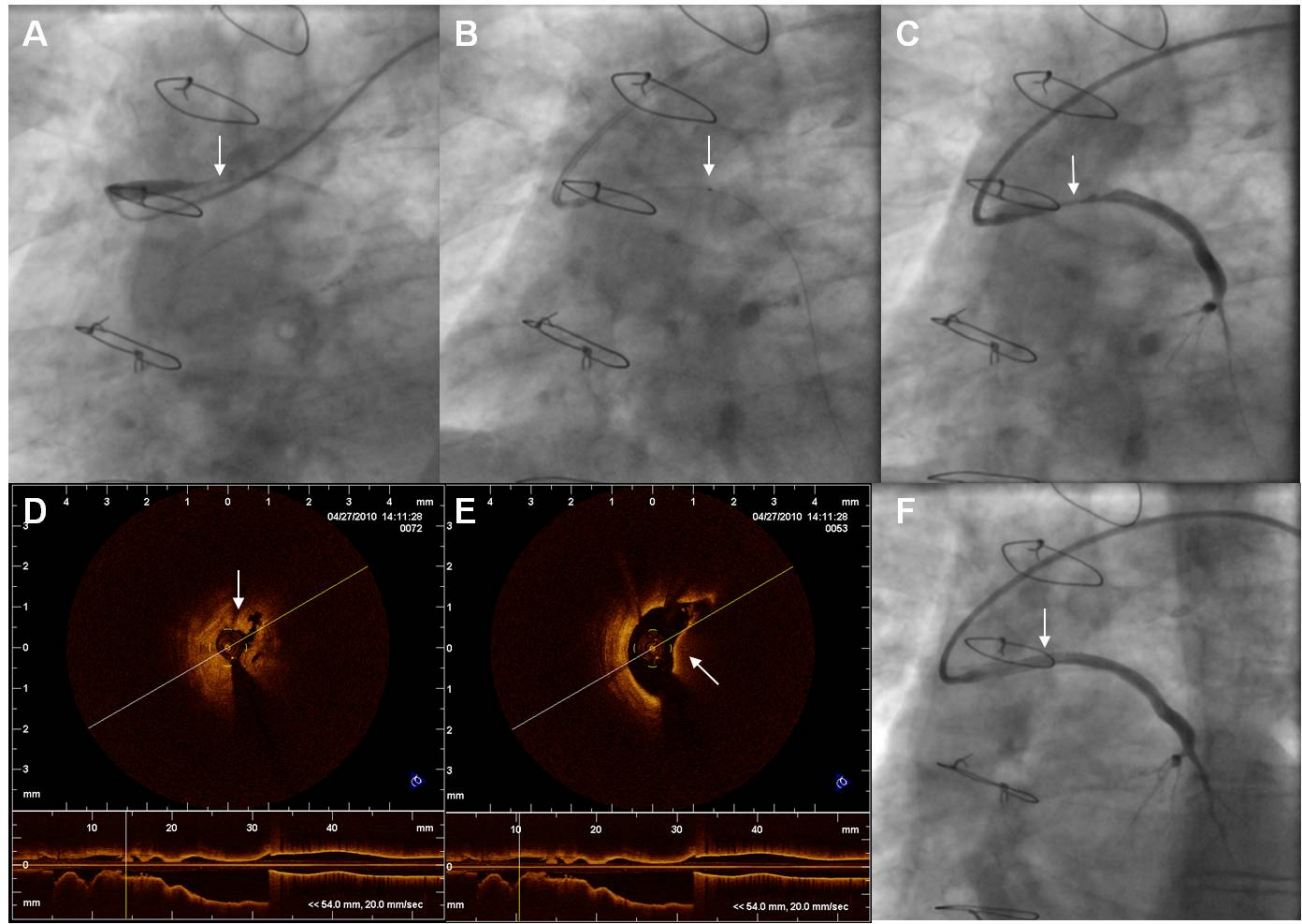
**Panel A:** An almost totally occluded SVG to LAD (arrow), in a patient with an anterior STEMI. **Panel B:** Advancement of the thrombus aspiration device (Export, Medronic Vascular, USA) through the lesion. Arrow points to the distal radiopaque tip marker of the aspiration device. **Panel C:** Post thromboaspiration the SVG lesion (arrow) and the full length of the SVG to LAD is visualized. **Panel D:** OCT imaging. Arrow points to white thrombus at the level of the narrowest point. **Panel E:** OCT imaging. Arrow points to red thrombus. **Panel F:** Final angiographic result following stenting (arrow). LAD: Left Anterior Descending artery, OCT: Optical Coherence Tomography.

**Fig. (5):**
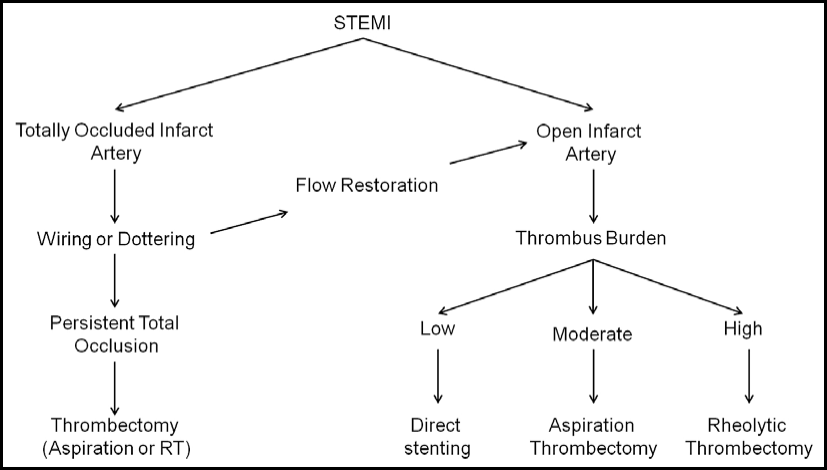
Proposed algorithm for the use of thrombectomy devices in patients with STEMI.

Angioplasty of Saphenous Vein Grafts (SVGs), especially when there is total occlusion, has poor long-term results compared to native vessels PCI [6, 32-34]. Suboptimal results have been attributed to the larger TB and the large/friable plaque mass of old SVGs, leading to distal embolization/no-reflow [33, 35]. In our experience, visible angiographic thrombus is present in approximately 20% of ACS patients with culprit old SVG lesions [36]. However, using optical coherence tomography, thrombus was detected in 46% of such lesions, being significantly more frequent in patients with STEMI [36]. (Fig. **4**). A number of treatment options are available for SVG-PCI in the setting of ACS (including acute occlusion), such as covered stents, distal filter protection (DFP), and thrombectomy. Although DFP is a Class-I indication for all SVG-PCI to prevent distal embolization [6], DFP devices may not be easily and/or safely used in occluded SVGs, or in SVGs perfusing more than one native vessels. Additionally, large TB and friable tissue may cause filter overloading [35]. Although thrombectomy might be an alternative, there are no large studies examining the potential benefit of the latter in SVG-PCI. Despite this, in some centers thrombectomy has been reported to be the main adjunctive technique involved for contemporary PCI of old acutely occluded SVGs, with good results [33].

Thrombus aspiration is relatively easy in SVGs due to their large caliper, and has been successfully performed even with the guiding catheter [37]. As DFP constitutes the standard of care for SVG-PCI [6], when dealing with thrombotic SVGs, combination with thrombectomy seems attractive. This concept is not new, as one distal protection device involving an occlusive balloon (PercuSurge GuardWire, *Medtronic AVE, Minneapolis, Minnesota*), allows for AT [38]. Its use during stenting in diseased SVGs reduces periprocedural complications [39]. However there is no agreement as to whether this system may induce balloon injury on the vein graft wall when visible atherosclerotic disease is present.[40, 41] As there is no available device combining AT with nonocclusive DFP, sequential use of two such devices has been proposed. This strategy has been tested in patients at high risk for distal embolization in both native coronaries and SVGs, and found to be feasible and successful [35, 38]. However, in a series of 25 consecutive patients with SVG lesions treated with AT before stenting with the combined use of the FilterWire EX/EZ (Boston Scientific Corp., Natick, Massachusetts)**, **the incidence of no reflow was not reduced, compared to use of DFP alone [42]. Hence, the possible benefits of such a strategy should be prospectively tested taking into account the increased complexity of the procedure.

A high procedural success (91%) has been reported with RT in 72 patients with ACS (unstable angina, or MI) and angiographic thrombus, including 33 (46%) SVG interventions [43]. A multicentre study using X-SIZER, in 797 patients with 839 diseased SVGs or thrombus-containing native coronaries, found that this strategy did not reduce peri-procedural MI or MACES at 30 days and 1 year compared to PCI alone, although the rate of large MI was reduced [44]. In contrast, both balloon occlusion/aspiration and DFP devices reduced the incidence of small and large MI. Finally, cases of successful combination of RT with the PercuSurge distal balloon occlusion device have been reported [45]. In general, both AT and MT and their combination with DFP are feasible, and may be useful in the management of highly thrombotic infarct-related arteries/SVGs, however proper testing of this hypothesis is needed [38].

## CONCLUSION

Aspiration thrombectomy, as an adjunct to PCI in thrombotic native lesions (mainly during pPCI), is effective and safe, and could possibly benefit the most patients with a large TB. New evidence also suggests that RT might be beneficial for such patients. Sequential application of manual/mechanical thrombectomy and DFP might be a reasonable approach for thrombotic SVG lesions, however robust evidence is still lacking.
